# Understanding the link between interstitial lung disease and obstructive sleep apnea: is lung volume involved?

**DOI:** 10.36416/1806-3756/e20240356

**Published:** 2024-11-16

**Authors:** Pedro Rodrigues Genta, Paulo Mateus Madureira Soares Mariano

**Affiliations:** 1. Unidade de Disturbios do Sono, Divisao de Pneumologia, Instituto do Coracao (InCor), Hospital das Clinicas HCFMUSP, Faculdade de Medicina, Universidade de Sao Paulo, Sao Paulo, SP, BR.

Obstructive sleep apnea (OSA) is associated with unfavorable cardiovascular, metabolic, and neurocognitive outcomes, resulting in significant deterioration in quality of life and increased mortality.^1^ The coexistence of interstitial lung disease (ILD) and OSA represents an additional, potentially harmful combination.^2^ ^-^ ^4^ Previous small studies assessed OSA in patients with idiopathic pulmonary fibrosis (IPF).^5^ ^-^ ^7^ In this issue of the JBP, Cardoso et al. investigated the association between non-IPF ILD and OSA.^8^ The relevance of the study and the contribution of the authors are emphasized, given the scarcity of data on this comorbidity. In addition, the authors provide additional evidence of the effect of CPAP in patients with both OSA and ILD.

In agreement with previous studies, the results by Cardoso et al.^8^ highlight the limitations of anthropometric and demographic analyses and sleep questionnaires in the accurate identification of OSA in patients with ILD.^6^ ^,^ ^8^ ^-^ ^10^ A high OSA prevalence (76%) was reported,^8^ which is similar to the prevalence reported in a recent meta-analysis.^11^ The similar prevalence of OSA in the study by Cardoso et al.^8^ and in previous studies that mainly included patients with IPF suggests that the pathogenesis of OSA among both groups of patients is similar. Patients with OSA had significantly lower total lung capacity (TLC), suggesting TLC as a predictor of OSA risk.^8^ A TLC < 80% of predicted was associated with an 82% probability of OSA.^8^ Reduced lung volume may partially explain the high prevalence of OSA.^12^ ^-^ ^15^ A lower lung volume may increase upper airway collapsibility due to reduced tracheal traction.^12^ ^-^ ^14^ The high prevalence of OSA is also potentially attributable to the patients’ age, as increasing age is an important risk factor for OSA.^16^ Future studies that compare the prevalence of OSA among ILD patients and a control group consisting of patients without ILD of similar age, gender, and BMI are necessary to determine if ILD is associated with increased OSA prevalence.

Previous studies suggested that OSA is associated with clinical deterioration and reduced survival.^2^ ^-^ ^4^ The majority of OSA patients in the study by Cardoso et al.^8^ were not previously recognized. Underecognition of OSA precludes treatment and potentially leads to impaired quality of life and poorer prognosis. In the study by Cardoso et al., 12 patients underwent CPAP treatment.^8^ After three months, 91% demonstrated effective control of OSA, with improvement in daytime sleepiness and a tendency to improve emotional well-being.^8^ A previous observational study that included 55 subjects suggested that CPAP treatment improved survival.^3^ However, a randomized controlled trial to test the benefit of CPAP among patients with ILD and OSA is necessary before active screening and treatment of OSA are recommended.

In addition to the lack of a control group and the observational nature of the study, the authors acknowledged some additional limitations of their study. The sample was relatively small and came from a single center, which restricts the overall applicability of the results and their representativeness in different clinical settings. In addition, adherence to PAP therapy was under optimal (66%). Low adherence to CPAP is a common feature in previous studies and may compromise the potential long-term benefits of the treatment.^17^ The heterogeneity of the lung disease across patients may also influence the results, due to variations in the evolution and response to treatment between the different fibrotic conditions.

The findings of the study conducted by Cardoso et al.^8^ may influence medical practice. They emphasize the need for further investigation of sleep disorders in patients with ILD. These patients may benefit from early screening, especially when associated with reduced TLC. The study also demonstrates that PAP treatment in patients with ILD may improve quality of life. Controlled trials are necessary to determine the impact of OSA treatment among patients with ILD.
